# Explaining the adaptation gap in Dutch coastal risk management through lock-in mechanisms

**DOI:** 10.1007/s10113-025-02411-y

**Published:** 2025-06-13

**Authors:** Bram De Botselier, Lisanne Groen, Jean Hugé, Dave Huitema

**Affiliations:** 1 Flemish Energy and Climate Agency, Brussels, Belgium; 2 Department of Environmental Sciences, Open Universiteit, Heerlen, The Netherlands; 3 Brussels School of Governance, Vrije Universiteit Brussel, Brussels, Belgium; 4 Systems Ecology & Resource Management Unit, Biology Department, Université Libre de Bruxelles (ULB), Brussels, Belgium; 5 Plant Ecology & Nature Management, Biology Department, Vrije Universiteit Brussel (VUB), Brussels, Belgium; 6 Zoology: Biodiversity & Toxicology, Centre for Environmental Science, Hasselt University, Hasselt, Belgium; 7 Public Administration and Policy Group, Wageningen University, Wageningen, The Netherlands; 8 Marine Biology Research Group, Biology Department, Ghent University, Ghent, Belgium

**Keywords:** Coastal management, Delta Programme, Climate change adaptation, Lock-ins, Climate adaptation gap, The Netherlands

## Abstract

This article investigates Dutch coastal risk management in light of recent scientific evidence on long-term sea level rise. While the so-called *Delta Programme*, meant as a national boost for flood safety, remains central to the Netherlands’ coastal defence strategy, our analysis reveals that it does not offer sufficient protection beyond the year 2050. It is therefore evidence of a climate adaptation gap. Drawing on the concept of lock-ins, this study examines how certain mechanisms may be at play. The study uses a framework on “adaptation lock-ins” developed by Groen et al. (2022) in an attempt to study and explain this adaptation gap. A qualitative analysis of policy documents, secondary literature and semi-structured interviews with experts points to the existence of several lock-in mechanisms which are implied in the stagnation of Dutch coastal risk management. The insights gathered are relevant for practitioners and academics alike as it might be used to inform the upcoming revision of the *Delta Programme* in 2027. At the conceptual level, the study adds insights on previously undiscovered types of lock-ins, thus adding to the explanatory power of the lock-in concept. The article concludes that further research can focus on how lock-ins vary across time, regions or contexts, and how they can be addressed and overcome.

## Introduction

As a result of the increase in global mean surface temperature, the global mean sea level rise will likely reach 1 meter (m) by 2100 (Intergovernmental Panel on Climate Change (IPCC) 2021). However, this effect is subject to regional variation, and if certain tipping points are reached (see e.g. Armstrong McKay et al. 2022), sea levels might rise more than currently foreseen. Bamber et al. (2019) concluded that global mean sea level rise of more than 2 m by 2100 is possible in case of a high emissions scenario.

Here, we are interested in the Netherlands. Due to regional conditions and Antarctic melt, sea level rise alongside the Dutch coast is expected to be higher than the global mean (Haasnoot et al. 2018). Sea level rise along the Dutch coast could reach 1.2 m by 2100 in a high emissions scenario, and over 2 m if Antarctic melting accelerates (Dutch Meteorological Institute (KNMI) 2021). The IPCC’s recommendation to consider more extreme sea level rise scenarios than the likely scenario of 1 m sea level rise by 2100 is thus particularly salient for the Netherlands (Fox-Kemper et al. 2021; IPCC 2021), also because centuries of land reclamation have resulted in 55% of Dutch territory and 62% of its urban area embanked (Schultz Van Haegen and Wieriks 2015). Haasnoot and Diermanse (2022) therefore recommend that Dutch planning scenarios account for up to 2–3 m of sea level rise. Strategic choices on the future of Dutch coastal management have to be made, potentially with far-reaching consequences, and no-regret measures, which are useful no matter which future policies are implemented, should be taken. It specifically concerns for example spatial reservation for future flood defences, water discharge or storage (Van Alphen et al. 2022). Nonetheless, Haasnoot et al. (2018) note that incumbent Dutch policies, based on a maximum 1 m rise by 2100, underestimate future risks. They argue that many existing coastal defences could become inadequate beyond 2050.

This is surprising given the Netherlands’ international reputation in flood management, largely shaped by the Delta Works programme. This building programme led to a series of dams, dykes and storm surge barriers constructed through 1997, that are still in use today. The aim of the Delta Works was to shorten the coastline and consequently protect the Netherlands in storm conditions. The construction started based on recommendations of the Delta Commission, an expert commission established on 18 February 1953 in response to a severe flood in January 1953 (Schultz Van Haegen and Wieriks 2015). To address future challenges, a second Delta Commission was established in 2007, leading to the launch of the Delta Programme in 2011. This programme coordinates planning related to flood safety, freshwater availability and sea level rise in response to long-term (defined as “until 2100 and after”) climate change between the different levels of government involved. The national government, provinces, municipalities and water boards all participate. Water boards are local governing bodies dedicated to water management. Different steering groups and regional groupings exist within the Delta Programme, as well as specific thematic programmes. The most relevant for this study are the general steering group on water safety, the National Consultation Coast and the Flood Protection Programme. The Delta Fund ensures stable, long-term financing outside of the annual budgetary negotiations. The 2017 Water Act, which sets protection standards for primary flood defences by 2050, guides much of the current policy focus (Schultz Van Haegen and Wieriks 2015; Van Alphen 2016).

The Delta Programme operates through adaptive management, with 6-year evaluation cycles. Despite new insights (e.g. Haasnoot et al. 2018), the 2021 policy review introduced no significant changes to guidance for the 2050 horizon, underscoring a continued emphasis on protecting the coastline in its current state. Alternatives such as increasing resilience of vulnerable areas (accommodation), inland migration (retreat) or using a seaward strategy (attack) are wholly absent in the plans (Dedekorkut-Howes et al. 2020; Dronkers et al. 1990; Haasnoot et al. 2019). A so-called adaptation gap, namely a difference between the level of adaptation needed in light of long-term climate change, and adaptation policies that are currently in place, can thus be observed in Dutch coastal risk management policy (UNEP 2023).

So why have more extreme sea level rise scenarios not been considered in Dutch coastal risk management? The persistence of the current approach despite scientific evidence that it is not suited in light of long-term sea level rise remains underexplored. This article builds on emerging research applying lock-in perspectives to climate adaptation (Groen et al. 2022; Jager, King and Siebenhüner), and applies it to Dutch coastal risk management.

Studies on Dutch flood defence policy can already provide some initial insights. Generally speaking, researchers noted a relatively stable policy development with high levels of path dependency and incremental change (e.g. Kaufmann et al. 2016; Meijerink 2005) with flood events as an important booster for policy change (e.g. Huitema and Meijerink 2009; Kaufmann et al. 2016; Verduijn et al. 2012; Zegwaard et al. 2015). Other studies have specifically aimed to analyse one or more (f)actors that boost or hinder policy progress on climate adaptation in Dutch water management. Bloemen et al. (2019) looked into the development of climate adaptation policy in the early 2000s and pointed towards bureaucratic struggles as a stumbling block, in particular between the ministry responsible for the environment, also in charge of adaptation policy, and the ministry responsible for water management. Policy progress therefore required these struggles to be overcome. Biesbroek and Candel (2020) concluded that fragmentation in national decision-making and a lack of coordination may also lead to delays. Schultz Van Haegen and Wieriks (2015) and Van Alphen (2016) specifically discussed the link between flood safety and climate adaptation with a study of the Delta Programme, focusing on institutional aspects, such as the role of the Delta Commissioner as a factor of stability throughout several changes in government.

Several of the studies mentioned above fit with a strand of literature that considers a lack of policy progress to be due to the absence of policy dynamics and as the result of specific barriers. However, it has since then been demonstrated that policy stability is often the result of a dynamic process. This idea is reflected in the concept of lock-in, which has been used in political science and economics literature. More recently, the concept of lock-in has also been applied to provide useful insights when explaining policy stability in climate adaptation policy. Groen et al. (2022) and Jager et al. (2022) have drawn on previous literature to study coastal adaptation in Germany and the UK from a lock-in perspective. Building on Groen et al. (2022) in particular, climate adaptation policy lock-in can be defined as a set of mutually or self-reinforcing mechanisms which maintain incumbent coastal risk management policy, preventing more extreme scenarios of sea level rise from being sufficiently taken into account.

Despite the tradition of (academic research on) flood safety, research on adaptation to long-term climate change in the Netherlands is still relatively new. Additionally, Dutch coastal risk management has not yet been studied from a lock-in perspective. This contribution therefore draws on the research by Groen et al. (2022) and Jager et al. (2022) and uses a similar perspective for the Netherlands. It aims to look into the workings of (and interaction between) the different mechanisms that hinder policy making in line with climate adaptation needs in the field of coastal risk management in the Netherlands, and specifically asks the following research question: to what extent can taking a lock-in perspective explain the adaptation gap in Dutch coastal risk management, and which lock-in mechanisms are involved?

This article first elaborates on the concept of climate adaptation lock-ins; then describes the methods used; subsequently expands on the empirical findings; then links the findings to the analytical framework in the discussion section before concluding. As such, the article contributes to the literature in different ways. First, this article is an addition to the literature on Dutch water management and climate adaptation, as it provides an in-depth analysis of Dutch coastal risk management from a perspective of adaptation to long-term climate change. Its insights can be relevant for the next major review of the current strategies that is foreseen in the 2027 Delta Programme (Deltaprogramma 2023). Second, the article contributes to the literature on lock-in mechanisms, and the new strand of studies that employ this concept to climate adaptation. As such, it can provide additional empirical evidence for lock-in mechanisms that have also been observed in other case studies, or, alternatively, suggest different lock-in mechanisms that could also be relevant.

## Identifying climate adaptation lock-ins

The lock-in literature generally divides lock-ins into three categories, namely institutional, technological and behavioural (Groen et al. 2022; Jager et al. 2022; Kotilainen et al. 2019). Institutional lock-in mechanisms are influenced by the functioning of institutions, organisational practices, social norms and politics. Technological or infrastructural lock-in mechanisms are the result of incumbent technologies and existing infrastructure impacting current policy. They may arise due to sunk costs, as investments made in the past influence behaviour and investments in the future (Gifford 2011). Behavioural lock-in mechanisms are influenced by societal customs, habits and traditions, and exist due to a preference to stick to what is familiar (Kotilainen et al. 2019; Zauberman 2003). Other lock-in categories have been identified in the literature, such as lock-ins related to discourse, knowledge and actors (e.g. Siebenhüner et al. 2021). Since this study focuses on individual lock-in mechanisms and their interaction, rather than on the division into specific categories, we do not discuss the categorisation further.

Even though lock-ins had not yet been used to study climate adaptation gaps, Groen et al. (2022) and Jager et al. (2022) were able to build on scholars that studied path dependency and policy stability and contributed to the development of lock-ins as a concept. They drew for instance on Arthur and Arrow (1994), who built on economic arguments and focused on lock-ins created by technology, whereas Pierson (2000) and Foxon (2002) also looked into the role of institutions. Zauberman (2003) explained how consumer behaviour contributes to the creation and continuation of lock-ins. Under the term carbon lock-in and first developed by Unruh (2000), the lock-in concept has been used to study climate mitigation. Klitkou et al. (2015) and Kotilainen et al. (2019) for instance used this approach to study lock-ins hindering the energy transition and the roll-out of sustainable mobility, respectively.

Groen et al. (2022) consequently identified lock-in mechanisms that have been well documented in the literature. These lock-ins, as defined by Groen et al. (2022), will also serve as the analytical framework for this study.

Collective action refers to the role of key stakeholders. If the framing of the issue by stakeholders and the interests they promote result in pressure to continue current coastal risk management, collective action can constitute a lock-in mechanism. Power differentiation refers to the push for a continuation of existing policies by actors in power in order to strengthen their own position, thereby creating a lock-in (Klitkou et al. 2015).

Spreading the cost of earlier investments over a larger number of units of the same (economies of scale) or a similar (economies of scope) technology lowers the cost per unit. This may seem like a cost-saving measure, but leads to alternative solutions being left out of consideration, and can thus constitute a lock-in mechanism (Kotilainen et al. 2019).

Adaptive expectations refer to how institutions, organisations or individuals can adjust their behaviour based on how they perceive that others will act (Pierson 2000). This can constitute a lock-in when the continuation of current policies is anticipated, leading to continued asset accumulation in vulnerable areas, which in turns results in additional pressure to keep existing policies in place. Learning effects lead to a lock-in if knowledge and experience from existing coastal defences result in a focus on improving these defences, rather than considering alternatives (Groen et al. 2022; Klitkou et al. 2015; Kotilainen et al. 2019). Some studies distinguish between technological and institutional learning effects, but the government, and especially the executive agency Rijkswaterstaat, is a key player in regulating, designing and implementing measures in Dutch coastal risk management, rendering this distinction less relevant in our case.

The habituation lock-in mechanism refers to the mental barrier hindering consideration of alternative coastal defences due to citizens’ familiarity with, confidence in and subsequent attachment to existing structures. This can lead to a lock-in, for example if the education of different generations of engineers is limited to what is currently known, without challenging the status quo. Also future generations of engineers will then be educated with the same knowledge (Kotilainen et al. 2019; Murray and Häubl 2007; Zauberman 2003).

While the different lock-in mechanisms have been introduced individually, it is important to note that they may be interconnected or overlapping. However, in order to allow for disentangling the different underlying dynamics, it has merit to analyse the different mechanisms individually as is done in the “Discussion” section. Overlaps and interconnections as well as mutually and self-reinforcing effects are mentioned wherever relevant.

## Methods

The analysis is based on a qualitative, in-depth single case study using policy documents, secondary literature and semi-structured interviews. It applies the framework by Groen et al. (2022), as discussed in the previous section, to a new case. By applying a clear and pre-established framework, we hope to advance the understanding of lock-ins, the dynamics implied in their creation and maintenance, and their interactions in the field of climate adaptation. A process tracing approach was adopted to systematically link historical policy developments to observed lock-in mechanisms.

### Data collection

Three types of data were used for our empirical research: policy documents, and particularly the Delta Programmes (editions 2011 until 2024, published between 2010 and 2023) and related government decisions, such as the National Water Plans of 2016–2021 and 2022–2027; existing secondary literature about the set-up of the Delta Programme (see e.g. Bloemen et al. 2019; Huitema and Meijerink 2009; Kaufmann et al. 2016; Schultz Van Haegen and Wieriks 2015; Van Alphen 2016; Verduijn et al. 2012; Zegwaard et al. 2015); and 10 semi-structured interviews with 13 experts (Open Universiteit Nederland Research Ethics Committee cETO approval number U202006491).

Interviewees were largely selected based on their roles in different organisations active in water management at the time, as well as based on previous relevant experience. Some experts were contacted via the snowball technique (Bryman 2016). Additionally, it was ensured that a wide range of expertise was included, such as professionals from academia, as well as from the private and public sector, with different levels of government represented among the interviewees. With the exception of interview 6, all interviews were conducted with senior experts with on average 19 years of experience in Dutch water management, coastal policy and spatial development. An overview of the different interviewees is given in Table 1. The discussions with the experts followed a semi-structured approach and typically lasted around 1 h. Questions addressed the expert’s view on how climate adaptation is incorporated in coastal risk management, how this has evolved over time, which (f)actors, dynamics and mechanisms had an impact on this evolution, and which obstacles remain. The interviewees were informed about the research purpose and signed the appropriate consent forms.

**Table 1:** Overview of interviews (source: compiled by authors)

Interview number	Interview date	Employment
1	8 December 2020	Government (national)
2	23 February 2021	Staff Delta Commissioner
3	22 March 2021	Government (provincial)
4	30 March 2021	Government (water board)
5	2 April 2021	Government (water board)
6	12 April 2021	Government (water board)
7	13 April 2021	Private consultant
8	23 April 2021	Government (national)
9	4 August 2021	Research institute
10	6 August 2021	Staff Delta Commissioner

### Data analysis

In our analysis of the policy documents, we focused on identifying key themes and patterns through a close reading, without applying formal coding procedures. Secondary literature was used to gain a better understanding of the policy dynamics at the time of the Delta Programme’s establishment. This allowed us to identify potential trends and possible factors influencing policy development, nevertheless stopping short of establishing clear causal links.

Additionally, the interviews were transcribed based on recordings. A coding protocol was developed and used for the analysis of the transcripts, in order to ensure consistent coding. The coding protocol was designed in a deductive manner, departing from the lock-in dimensions identified in the analytical framework above, in order to identify which lock-in mechanisms may explain the adaptation gap in Dutch coastal risk management. Additionally, the coding protocol also allowed for identifying additional lock-in mechanisms in an inductive manner. The coding process was facilitated using Atlas.ti software.

We used 2023 as a baseline and then used process tracing techniques in order to trace back the origins and underlying reasons for certain policy choices and subsequent development and to identify whether these could be linked to lock-in mechanisms. Process tracing is particularly useful for unpacking complex causal mechanisms in a single case, as is the case in this article. Since process tracing is heavily reliant on the availability and interpretation of qualitative data (Beach and Pedersen 2016), this limits the generalisability of our findings.

## Findings: identifying lock-in mechanisms in Dutch coastal risk management

### Technological first mover disadvantage

Current Dutch coastal risk management is based on a protection-based strategy, focused on protecting and maintaining the coastline in its current state, in order to safeguard economic assets and citizens in low lying regions. At the centre of this policy is the principle of “soft wherever possible, hard wherever necessary”, which was first promoted by the National Coastal Vision of 2013 (Deltaprogramma 2013). The preferred policy option is to keep the Dutch natural coastal defences, namely beaches and dunes, intact. This principle has resulted in a heavy focus on sand nourishment, which is needed to maintain natural barriers and compensate for natural processes such as erosion. When this is not possible, for example due to the absence of natural barriers, hard infrastructure such as dykes is considered (Deltaprogramma 2014). Both sand nourishment and hard infrastructure are protection-based and resource-intensive approaches, which are not well-suited in light of long-term and more extreme sea level rise alongside the Dutch coast (Haasnoot et al. 2018). Alternative approaches, such as increasing resilience of vulnerable areas (accommodation), inland migration (retreat) or using a seaward strategy (attack), are not covered by this basic principle of Dutch policy (Dedekorkut-Howes et al. 2020).

Since the relative cost of sand nourishment decreases as the volume increases, there is an incentive to use sand nourishment on a larger scale in order to reduce relative costs, rather than considering alternative options. This is an example of a lock-in due to economies of scale (Interview 9). Additionally, when asked about innovation in Dutch coastal risk management, the Sand Engine was referred to as an example by five interviewees. The Sand Engine is a coastal defence project that included a one-time large-scale nourishment through the creation of an artificial sand bank, replacing the traditional, frequent sand nourishments. The sand is carried to the coast via natural currents, indirectly resulting in sand nourishment. The Sand Engine’s first official evaluation in 2021 was largely positive (Gerdes et al. 2021). The Sand Engine project exemplifies innovation within the existing paradigm, improving the efficiency of protection-based strategies rather than challenging them. It shows that new initiatives are limited to making more efficient use of existing technologies by applying them to a broader range of coastal defences, in an attempt to increase cost effectiveness. It does not prepare for more extreme scenarios of sea level rise (Haasnoot et al. 2018; Interview 8). This is an example of the lock-in mechanism economies of scope.

A similar observation can be made for innovative examples of hard defences given by the interviewees, such as the Hondsbossche sea wall and the Katwijk coastal defence. The Hondsbossche sea wall is innovative in the sense that it combines a hard structure with sand nourishment, allowing for a combination of flood protection and recreation. The coastal construction in Katwijk includes a parking garage and is thus also multi-purpose. However, these examples cannot be considered a departure from the existing approach or as better suited for more extreme sea level rise. Rather, they fit with the protection-based approach and benefit from spill-over effects of existing mechanisms (Dedekorkut-Howes et al. 2020). This can be seen as a lock-in due to economies of scope, because the focus is on improving existing technologies in an attempt to increase cost effectiveness, rather than on designing technologies better suited for long-term sea level rise.

Whereas the above paragraphs describe how costs can be lowered by spreading initial investments over more units, costs can also be lowered by increasing skills and efficiency through learning by doing, leading to a possible learning effects lock-in mechanism. Learning effects were consciously used during the Delta Works. The smallest storm surge barrier was constructed first and the experience was used later for the construction of the larger ones (Disco 2002). This points towards learning effects being intrinsic to the development of Dutch coastal policy. Through the years, Rijkswaterstaat has indeed gained significant experience when it comes to sand nourishment, which has according to one interviewee resulted in an “almost blind faith” in Rijkswaterstaat’s expertise, so much so that sand nourishment has consequently become “the go-to choice” (Interview 2). A similar phenomenon exists with regard to the human-made, hard coastal defences. At the time of their construction as part of the Delta Works, the Dutch storm surge barriers were very innovative. A generation of officials at Rijkswaterstaat has since gained significant experience in maintaining and operating the existing systems and has become very good at it (Interview 4). These elements indicate that learning effects indeed play a role in explaining the adaptation gap. The long-standing success of protection-based approaches, such as dike construction and reinforcement, has led to a wealth of experience and expertise in these methods. This accumulated knowledge makes such approaches appear more reliable and cost-effective compared to untested alternatives, thereby creating a preference for the status quo. The entrenched learning effects make it challenging to shift towards alternative strategies, which lack the same level of institutional support and perceived reliability.

Economies of scale and scope and learning effects are not only self-reinforcing mechanisms, but also strengthen each other, since they all decrease the cost of continuing existing policies. Through its long tradition of water management, the Netherlands may have become so efficient at sand nourishment and hard coastal defences that there is little economic incentive to consider alternative coastal defence strategies, even though they may be more suitable for the long term.

### Influencing behaviour: the Netherlands as a victim of its own success?

When confronted with new policy proposals, citizens and other stakeholders tend to make a cost–benefit analysis of what the change would mean for them (Kotilainen et al. 2019; Murray and Häubl 2007; Zauberman 2003). In the case of coastal risk management, this cost–benefit calculationcan be an important barrier to policy change, since the costs and benefits of switching to a new policy take place asynchronously. Interviewees mention that the costs are incurred in the short term, namely giving up land or investing in flexible building methods. The cost is also perceived to be very high, among others due to the sentiments of the disastrous 1953 flood, which has created a mentality that the seawater should be kept away from land by any means necessary. This memory makes it more difficult to discuss alternative policies, such as giving land back to the sea, because they are perceived to include a higher risk compared to incumbent policies. In contrast to the (perceived) risks of adapting to it, extreme sea level rise itself is not yet visible alongside the Dutch coast, meaning that it is seen as less serious. As a result, the benefits of responding to it will only become clear in the longer term (Interviews 4, 7 and 8). This narrative is also present in Rijkswaterstaat: in the section on challenges in the twenty-first century, its own website sees water management as a “battle against water [that] has not yet been won” (Rijkswaterstaat 2024). This framing leaves little room for alternative policies.

This further reinforces citizens’ confidence in current policies. The Netherlands’ historical relationship with the sea has created a high confidence in the human ability to shape the water environment, which has in turn resulted in a certain national pride. This is an example of habituationand is for example concretely visible in different political party programmes for the Dutch parliamentary elections in 2023, where references to the Delta Works are made to underline the Netherlands’ general potential and as a “golden standard” that also other policies should strive towards. The pride is thus inherently connected to the protection-based human-made character of the coastal defences. Citizens and decision-makers have grown accustomed to and have confidence in the current coastal risk management policies and the existing coastal defences. The trust in alternatives is (much) less high (Interviews 1, 3, 4, 7 and 8).

Habituation fosters a false sense of security regarding the risks of sea level rise beyond 2050. The presence of major defences seemingly reduces the urgency to address more extreme future scenarios, for instance by promoting flexible building practices or establishing no-go zones in highly vulnerable areas. As a result, investments continue to flow into regions that are likely to face increasing risks over the long term (Hekman and Booister 2020). For example, the prospect of extreme sea level rise appears to have been largely absent from considerations surrounding the planned construction of two new nuclear power plants in the flood-prone province of Zeeland (Hensen, 29 November 2022). Spatial planning is shaped by the assumption that current levels of protection will endure, reinforcing ongoing development in vulnerable areas, which in turns creates pressure to continue the protection-based approach (Interview 10). This is an example of the adaptive expectations lock-in mechanism.

### Financial mechanisms and short-term political dynamics

When it comes to flood risk management, the Delta Fund is almost exclusively aimed towards upgrading the Netherlands’ coastal defences to the standards of the 2017 Water Act. However, these standards are based on projections dating from the period 2009–2014, and focus on sea level rise in the period until 2050. They therefore do not consider more extreme sea level rise scenarios (Slootjes and van der Most 2016). The Delta Fund does in other words not finance measures for long-term sea level rise. Additionally, the Delta Fund’s strict funding mechanisms also make it difficult to combine it with other funding sources, which complicate the incorporation of coastal risk management considerations into ongoing projects. Four interviewees pointed to these elements of the Delta Fund’s set-up as a disincentive to investing in adaptation to long-term sea level rise (Interviews 4 and 6).

These strict funding rules are largely the result of the Delta Fund being established in 2008, during the beginning of the financial crisis. In times of budgetary constraints, the financial resources going to the Delta Fund were limited. As one interviewee closely involved at the time of its creation explained, even though it was understood that a broader focus of the Delta Fund would have been appropriate in light of the long-term challenges, due to the budgetary situation at the time it was preferred to have guaranteed financing for a Delta Fund that was more limited in scope (Interview 9).

The Delta Fund’s focus on short-term protection-based policies continues until today. The new coalition agreement (Rijksoverheid 2024) focuses narrowly on reinforcing dikes within the existing budgets, downgrading ambitions of the previous government to expand the Delta Fund budget and include more nature-based (albeit still largely protection-based) solutions (Rijksoverheid 2021). The focus on protection-based strategies is broadly supported across the political spectrum. A review of the eight biggest political parties’ (winning a combined 132 of 150 parliamentary seats) electoral programmes for the 2023 elections by the Union of Water Boards (16 November 2023) shows that the continuation of the current approach is supported by all main parties. Only two parties support somewhat alternative approaches, without providing substantial detail, while one did not mention coastal defence management at all.

This lock-in mechanism covering political decisions inspired by short-term electoral interests does not fully fit in the analytical framework developed by Groen et al. (2022). However, since it has contributed to the adaptation gap in Dutch coastal risk management, we will include it further in our discussion as the complexity and opacity of politics mechanism.

Five interviewees stressed that the Delta Fund could have been set up differently, referring to the Room for the River programme (2006–2019). This programme was set up in response to the 1993 and 1995 floods and had as its dual objective to reduce flood risk by accommodating a higher discharge capacity for rivers, and to increase the quality of spatial development. The latter allowed nature and recreational objectives to be incorporated and co-benefits and local concerns to be taken on board. The programme included a so-called replacement decision, which meant that local governments could propose alternative projects to the measures suggested by the national government, provided that the objectives were reached. Even though it was rarely used successfully, it provided an opportunity to engage with stakeholders and create a local support base for the projects (Andersson Elffers Felix 2013; Bötger and Beekmans 2017). The official, overall positive evaluation of the programme, as well as several interviewees, therefore consider Room for the River as a successful example of how flood safety and nature can go hand in hand (Interviews 1, 2, 5, 7, 10; Olde Wolbers et al. 2018; Zevenbergen et al. 2015).

The Delta Fund, in contrast, does not have the same flexibility to take local concerns on board. Whereas none of the interviewees pointed to local interest groups playing an important role in policy stability at present, this could change in the future. One interviewee mentioned that stakeholders could become much more involved if (national) decisions are taken, such as divesting away from vulnerable areas in light of long-term sea level rise, which would go against local interests (Interview 9). In the future, collective action could thus play a more important role as a lock-in mechanism, for which the Delta Fund is ill-prepared.

In one interview with three water board policy officers, it was mentioned that elected officials are sometimes afraid of being accused of fearmongering if they highlight the need to prepare for more extreme scenarios of sea level rise. From an electoral perspective, it can therefore be more interesting to be focused on short-term flood safety, which brings immediate results, rather than to promote alternative coastal risk management policies which only have benefits in the longer term (Interview 4). This indicates how the complexity and opacity of politics mechanisms continue to play a role in explaining policy stability, but also show how citizens’ confidence in the current strategy apparent through the habituationmechanism reinforces the complexity and opacity of politics mechanism.

### Decentralisation leading to responsibility avoidance

Another factor contributing to the adaptation gap referred to by four interviewees is the decentralised nature of spatial planning policy. The national government’s key role in spatial planning in the twentieth century, for example through the Delta Works, significantly weakened in the 1990 s due to decentralisation, and in particular the abolishment of the national ministry for spatial planning in 2010 (Interviews 3, 4, 5 and 8). Several reasons have been suggested to be at the basis for this decision, such as the ministry’s reputation for red tape among decision-makers and its attention for climate change. As such, it was an attractive, symbolic target for the new right-wing government. Additionally, the abolishment of the ministry fits the general austerity that was considered a political priority (König, 25 July 2022). This points towards complexity and opacity of politics being at the basis of the decentralisation trend.

Nowadays, local plans for spatial development, developed by municipalities and provinces, are the cornerstone of spatial planning in the Netherlands. However, these lower levels of government often have insufficient resources to take advantage of these competences, among others as a result of austerity. They also operate on a smaller scale, whereas coastal risk management requires a larger-scale approach. The national government can overrule the local plans if national interests are at stake, with flood safety as one of the key priorities. Despite this competence, however, the interviewees point out that the national government has limited influence and expertise when it comes to spatial planning (Interviews 3, 4, 5 and 8).

This has resulted in a situation where each level of government has a relatively narrow focus on its own key responsibilities, due to an inability (lack of resources or competences) and/or unwillingness (short-term nature of politics) to develop coastal risk management accommodating extreme sea level rise. This situation where the involvement of multiple actors leads to non-action, as described in this article, corresponds to what Groen et al. (2022) have identified as responsibility avoidance. They do not consider responsibility avoidance as a lock-in mechanism, but rather as a barrier, considering that it has no reinforcing effect. However, our analysis demonstrates that the decentralisation trend which resulted in responsibility avoidance was itself a consequence of the complexity and opacity of politics mechanism at play.

## Discussion

**Fig. 1:**
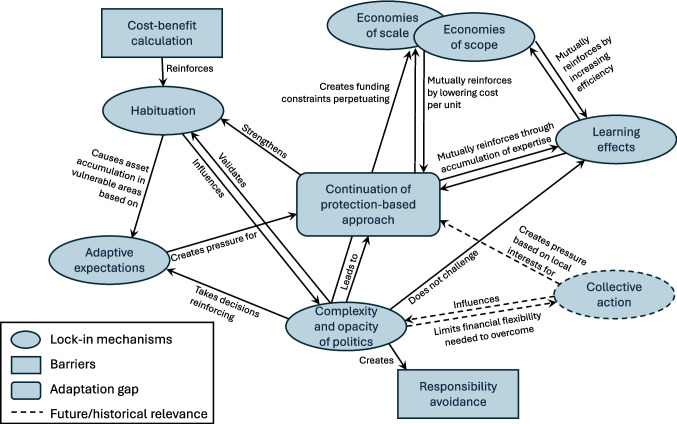
Lock-in mechanisms in Dutch coastal risk management (source: compiled by authors)

This study identifies several lock-in mechanisms that hinder Dutch policy responses to extreme sea level rise, as summarised in Fig. 1. In addition to these mechanisms, it also highlights two important barriers—responsibility avoidance and cost–benefit calculation—which, while not self-reinforcing and thus not lock-ins in the strict sense, are closely linked to habituation and the complexity and opacity of politics.

Our findings confirm that most lock-in mechanisms identified by Groen et al. (2022) are relevant in the Dutch context. As previously discussed, economies of scale, economies of scope and learning effects reinforce the continuation of the protection-based approach by making it appear cost-effective, even though it is poorly suited for long-term climate adaptation. Likewise, the Netherlands’ historical successes in water management have fostered strong habituation and adaptive expectations across society and politics. Citizens’ high trust in existing coastal defences encourages continued investments in vulnerable areas, further entrenching incumbent policies. The latest government agreement (Rijksoverheid 2024) does not suggest a policy shift, reaffirming reliance on dike reinforcements.

Moreover, the habituation mechanism is reinforced by the cost–benefit calculation barrier. Citizens tend to evaluate new policies through a short-term lens, perceiving the immediate costs of alternatives as outweighing long-term benefits. Psychological dynamics such as cognitive dissonance (Festinger 1957; Kuruppu and Liverman 2011) further inhibit behavioural change. Living in vulnerable areas while being aware of future risks creates discomfort, which citizens often resolve by downplaying the urgency of adaptation rather than by altering their behaviour. As previous research suggests (Verduijn et al. 2012; Zegwaard et al. 2015), extreme weather events can temporarily shift this calculation, because they highlight the hidden costs of policy inertia and open windows for change. This was also the case in the Room for the River programme, which was set up in response to the 1993 and 1995 floods of the Meuse and Rhine rivers.

In addition, short-term political considerations—particularly during the set-up of the Delta Fund—have constrained funding flexibility for long-term adaptation. Although this dynamic was not included in the analytical framework by Groen et al. (2022), it closely aligns with Pierson’s (2000) concept of the complexity and opacity of politics, which refers to politics as a complex environment, prone to the influence of outside stakeholders, outside events and short-term (often electoral) interests. Our findings show that this lock-in continues to shape Dutch coastal risk management today. Based on the priorities of the government that happens to be in office at the time of the Delta Programme’s major review in 2027, existing lock-in mechanisms such as adaptive expectations could be overcome, or conversely strengthened.

The funding dynamic through the Delta Fund reinforces other lock-in mechanisms. Similar to observations by Groen et al. (2022) in England and Schleswig–Holstein, the prioritisation of traditional protection-based projects perpetuates economies of scaleand scope, strengthens habituation by validating existing strategies and bolsters adaptive expectationsthat current approaches will persist, leading to continued investments in vulnerable areas.

Furthermore, the complexity and opacity of politics have led to a specific barrier: responsibility avoidance. Decentralisation of spatial planning responsibilities to provinces and municipalities, combined with austerity measures and the abolition of the national spatial planning ministry, has created a fragmented governance landscape. This situation of fragmented decision-making being a stumbling block for policy progress due to no single level of government being fully responsible for long-term adaptation has been identified in both Dutch (Biesbroek and Candel 2020) and English contexts (Groen et al. 2022).

Two lock-in mechanisms identified in the analytical framework do not play a major role in maintaining the adaptation gap. First, no empirical evidence was found for the existence of the power differentiation lock-in mechanism. In previous academic literature, this lock-in mechanism was found to play an important role in particular when certain private sector actors have an outsize influence on political decision-making, sometimes leading even to co-dependency if it results in a formal arrangement to continue current policies. Also for the less formal business network effects, which refers to policies created to suit private interests without necessarily direct lobbying from their side, no empirical evidence could be found in our case (Groen et al. 2022; Klitkou et al. 2015; Kotilainen et al. 2019). The absence of this effect could be explained by the central role of governmental actors Rijkswaterstaat and the Delta Commissioner in Dutch coastal risk management policy (Interviews 2 and 4). Additionally, key private actors involved in protection-based projects, such as dredging companies, are also involved in developing alternative strategies (Deltares 4 March 2024), making them less likely to oppose change.

Second, the role of the collective actionmechanism appears limited at present. Nevertheless, historically local interest groups have influenced Dutch flood risk management in favour of hard defences, thus contributing to the adaptation gap (Biesbroek et al. 2014; Disco 2002; Meijerink 2005; Interview 9). Different factors can explain why collective action plays a less important role at present. First, the dominant protection-based strategy faces little challenge, reducing incentives for collective action to protect local interests. Second, it is sometimes difficult to distinguish collective actionfrom the formal political process in the Netherlands. The Dutch consensus–based polder model originates from water management and the institutionalisation of civil society can still be observed today. Stakeholders for instance form political parties solely focused on participating in water board elections. One of the biggest political parties in water boards (Water Natuurlijk, or “Water Of Course”) is created by nature organisations (Havekes et al. 2017). As a result, many concerns from local interest groups can be captured through the formal political process. This dynamic could however change if decisions of national concern are taken that go against local interests. In this case, some interviewees expect the collective action mechanism to become more important again.

Through the identification of these lock-in mechanisms, this article contributes to a deeper understanding of the adaptation gap observed in Dutch coastal risk management. This knowledge can lead to the development of strategies to overcome the observed lock-in mechanisms, and eventually close the adaptation gap. It also highlights how the Netherlands’ long-standing tradition in water management may make some lock-in mechanisms more difficult to overcome, as also suggested by Jager et al. (2022).

It is useful to distinguish between passive and active forms of policy stability. Some lock-ins, such as habituation and adaptive expectations, are unintended products of past successes. Others, such as the complexity and opacity of politics and the reinforcement of economies of scale andscopethrough funding mechanisms, involve more conscious choices driven by short-term interests. The latter may be more amenable to intervention. Building on these insights, low-regret measures—such as limiting investments in vulnerable areas or reserving space for accommodation or seaward strategies (Van Alphen et al. 2022)—could gradually allow to overcome existing lock-ins. Broadening the Delta Fund’s mandate, inspired by the flexibility seen in the Room for the River programme, could also foster greater stakeholder support for transformational adaptation pathways.

Finally, it is worth noting that lock-in mechanisms are not static. As Kotilainen et al. (2019) argue, they may eventually be redirected to support alternative policies. Early projects exploring new adaptation strategies could, through learning effects and emerging economies of scale and scope, generate self-reinforcing dynamics that lower future costs and enhance political feasibility—thus laying the foundation for a shift away from the current protection-based paradigm. Specifically in the Netherlands for example, Room for the River has provided an opportunity to gain expertise with new approaches regarding river management, that could in turn lead to positive spillovers for alternative coastal risk management strategies through learning effects (Zevenbergen et al. 2015).

## Conclusion

This study examined the persistence of an adaptation gap in Dutch coastal risk management, despite mounting scientific evidence on the risks of extreme sea level rise. Although the Delta Programme remains central to the Netherlands’ flood defence strategy, no significant adjustments were made in the 2021 policy review, and alternatives such as increasing resilience of vulnerable areas (accommodation), inland migration (retreat) or using a seaward strategy (attack) remain absent. Given the Netherlands’ internationally renowned expertise in water management, this lack of adaptation is striking.

Building on a recent conceptualisation of climate adaptation lock-ins (Groen et al. 2022), this article analysed how different mechanisms reinforce the status quo. Using a qualitative analysis of policy documents, secondary literature and semi-structured expert interviews, it found that economies of scale and scope, learning effects, habituation, adaptive expectations, and the complexity and opacity of politics all contribute to maintaining the current protection-based approach. As a frontrunner in water management, the Netherlands is confronted with a first mover disadvantage due to its expertise with technologies that are not well-suited for long-term climate change. In addition, citizens and policy makers place high confidence in the incumbent strategy. Short-term political and electoral considerations, reflected in current government policies and funding mechanisms like the Delta Fund, further entrench existing strategies. Additionally, the study identified two important barriers—cost–benefit calculation and responsibility avoidance—that interact with these lock-ins, even though they are not self-reinforcing. Collective action, though historically significant, currently plays a limited role. However, this could change if future national decisions conflict with local priorities. Overall, the findings highlight the self- and mutually reinforcing nature of the different lock-in mechanisms.

The findings contribute to the literature in two ways. First, they add an in-depth case study of Dutch coastal risk management from a climate adaptation perspective, a field that remains relatively underexplored. Second, they extend the theoretical framework on lock-ins by emphasising the role of short-term political dynamics, such as electoral interests, which have so far received limited attention in the climate adaptation literature.

The study’s insights also have practical implications in light of the major review of the current strategies that is foreseen in the 2027 Delta Programme. While overcoming long-standing lock-ins may prove difficult, particularly given the country’s historical successes in water management, targeted interventions can loosen the lock-in dynamic. Measures such as limiting new investments in vulnerable areas, reserving space for alternative strategies and broadening the mandate of the Delta Fund could help shift the trajectory towards an adaptation strategy more suitable for long-term sea level rise.

Nevertheless, the study also acknowledges its limitations. First, the analysis is confined to only one case, the Netherlands, which limits the generalisability of the results to other contexts. Second, process tracing techniques were used to identify the historical roots of relevant policy decisions and their links to lock-in mechanisms. This approach has inherent limitations, such as its reliance on the availability and interpretation of qualitative data. In this study, while policy documents, secondary literature and expert interviews provided valuable insights, they primarily offered a retrospective account of events. This introduces the risk of bias and selective recall. Moreover, the lack of a fully longitudinal research design, including systematic data collection across a longer time span, restricts the ability to observe how lock-in mechanisms evolve or interact over time.

The study should therefore be seen as a comprehensive illustration of the underlying dynamics of the adaptation gap in Dutch coastal risk management. Future research could benefit from combining process tracing with a longitudinal approach to deepen the understanding of lock-in dynamics across time. Furthermore, the findings include several possible directions for future research, which could confirm the presence of the lock-in mechanisms identified, or delve deeper into the underlying dynamics and explore why certain mechanisms are more or less prominent in specific contexts. For example, in countries lacking a long-standing tradition of water management, lock-in mechanisms may be less pronounced or more easily surmountable. This could contribute to an understanding of how lock-in mechanisms can be addressed and overcome. A comparative approach may be particularly valuable in this regard. Additional research could approach the climate adaptation lock-in question from a more multidisciplinary perspective and combine adaptation literature with new insights from social psychology in order to further explain certain lock-in mechanisms, such as those related to behaviour.

## Data Availability

The interview transcripts generated during the study are available in the UK Data Service ReShare repository, record 856696. Additional data are available from the corresponding author upon reasonable request.
